# Toxic Features and Metabolomic Intervention of Glabrene, an Impurity Found in the Pharmaceutical Product of Glabridin

**DOI:** 10.3390/ijms25168985

**Published:** 2024-08-18

**Authors:** Xue Li, Haixin Jiang, Dongxue Guo, Wen Huang, Houpu Ren, Qiang Zhang

**Affiliations:** 1 Shaanxi Key Laboratory of Natural Products & Chemical Biology, College of Chemistry & Pharmacy, Northwest A&F University, Yangling 712100, China; leexue@nwafu.edu.cn (X.L.); 2544945256@nwafu.edu.cn (H.J.); xbnlgdx@nwafu.edu.cn (D.G.); huangwen0913@nwafu.edu.cn (W.H.); 2 College of Natural Resources and Environment, Northwest A&F University, Yangling 712100, China; renhoupu@nwafu.edu.cn

**Keywords:** glabrene, toxicity, phenylalanine metabolic pathway, *Glycyrrhiza glabra*, glabridin

## Abstract

Glabridin is a widely used product in the cosmetics and pharmaceutical industry, which is generally isolated and purified from Licorice (*Glycyrrhiza glabra*) extract in industrial production. It has wide clinical applications, but significant toxicity has also been reported. The purity of glabridin raw material is generally between 90% and 98%. We have identified a toxic impurity, glabrene, in the industrial product glabridin. Our investigation using an AB wild-type zebrafish toxicity test showed that glabrene has a significant lethal effect with an LC_10_ of 2.8 μM. Glabrene induced obvious malformation and disrupted cartilage development in zebrafish larvae. Furthermore, the compound significantly reduced larval mobility and caused damage to brain neural tissues. Metabolic pathway analysis and neurotransmitter quantification via ELISA indicated abnormal activation of the phenylalanine metabolic pathway, resulting in elevated dopamine and acetylcholine levels in vivo. These findings provide insights into the potential risks of glabrene contamination and offer a new reference point for enhancing safety measures and quality controls in licorice-derived products.

## 1. Introduction

**Figure 1:**
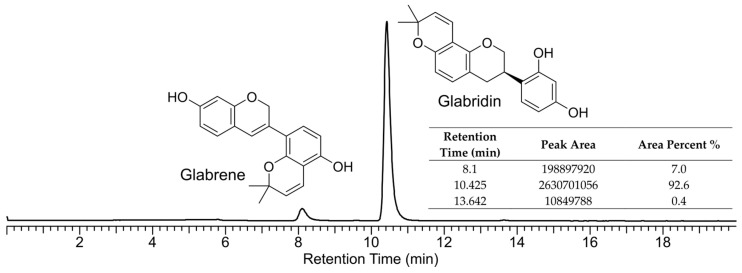
HPLC profile (254 nm) of the pharmaceutical product glabridin. The main impurity was glabrene.

Glabridin (Figure 1), a prenylated isoflavonoid, isolated from licorice (*Glycyrrhiza glabra* L.) roots, is known for its skin-whitening properties and commonly used in cosmetics for hyperpigmentation disorders [1]. It inhibits melanin synthesis by suppressing tyrosinase activity in melanoma cells, making it effective for treating hyperpigmentation [2]. Glabridin has been utilized in formulations designed to improve skin permeability and enhance the cosmetic appearance of melasma [3]. Glabridin exhibits a diverse range of pharmacological activities across various disease. For anti-cancer therapy, glabridin has played a dual role in enhancing the anti-metastatic potential of paclitaxel by impeding CYP2C8 in the liver and CYP2J2/EETs in tumor tissues of an orthotopic mouse model of breast cancer [4]. Recent metabolomic analyses have also revealed the crucial role of ERK in regulating metabolic pathways associated with the proliferation of human cutaneous T-cell lymphoma cells treated with glabridin. This finding underscores the potential of glabridin in targeting metabolic pathways in cancer treatment [5]. In autoimmune diseases, glabridin has immunomodulatory capabilities in Trex1-deficient mice [6]. Glabridin effectively reduces inflammatory responses by modulating specific inflammatory signals in both LPS-induced in vitro and in vivo models [7]. Moreover, glabridin has an antimicrobial property by inhibiting biofilm formation and swarming motility of multidrug-resistant *Acinetobacer baumannii* [8]. In pain management, glabridin therapy significantly reduces chronic allodynia, spinal microgliosis, and dendritic spine generation by inhibiting Fractalkine-CX3CR1 signaling in a mouse model of tibial fractures [9].

Various side effects have also been reported with glabridin products isolated from plants. Due to the presence of chiral carbon, the synthesis cost of glabridin is relatively high. The glabridin used in cosmetics is generally obtained through isolation from licorice (*G*. *glabra*) extract with a purity of 90-98%. The potential risks of using glabridin in the presence of impurities include increased toxicity levels [1,10]. Impurities, such as glabrol, have been shown to exacerbate the toxicity of glabridin in zebrafish embryos [1]. These impurities can introduce human safety risks, especially in consumer products like foods and cosmetics [2,11]. These risks include potential toxicity and allergenicity to the skin [2]. Additionally, inappropriate storage may cause licorice to produce certain mycotoxins, which are hazardous to humans [10]. Using licorice extracts may potentially present safety concerns due to unidentified impurities arising from the extraction or separation procedures undertaken [10]. The stability of glabridin is also a critical factor that influences its efficacy and application in therapeutic contexts. Several factors have been identified that affect the stability of glabridin, including temperature, illumination, humidity, pH, solvent, oxygen, and the presence of oxidants [12].

Such impurities can increase the risk of toxicity in glabridin products, highlighting the importance of controlling impurity levels during production. To minimize these risks, it is essential to control the impurities during the production of glabridin. Using biological evaluation techniques can provide more reliable data to assess the efficacy and biological activities of glabridin products, thereby supporting their clinical applications [13]. Additionally, the rigorous testing of samples for impurity content, like UPLC-Q-Exactive-Orbitrap-MS/MS analysis, can identify key impurities that contribute to toxicity [1]. Through monitoring and controlling impurity levels, the safety of glabridin products can be better ensured, thus minimizing potential risks associated with their use.

More importantly, searching for “dark” toxic substances in industrial products made from licorice is still essential to improve product quality and eliminate safety hazards. We have identified a typical impurity, glabrene, from the pharmaceutical or cosmetic raw materials of glabridin and revealed its significant acute toxicity. Glabrene is an isoflavone in licorice roots with two hydroxyl groups at positions 2′ and 7, a 2,2-dimethyl-γ-pyran ring fused to the B ring, and a double bond between C3 and C4 in the C ring, maximizing the double bond conjugation on the molecule [14]. Glabrene has estrogen-like effects [15] and is known for its cytotoxicity to cancer cells [16,17]. In addition, glabrene also plays a role in inhibiting tyrosinase-dependent melanin biosynthesis by inhibiting monophenol tyrosinase and bisphenolase tyrosinase activity [14]. In this process, glabrene exerts an inhibitory effect with a mixture of inhibitors and exhibits a dose-dependent effect. At the same time, because zebrafish skin and gills are relatively permeable to compounds diluted in the surrounding media [18], we further investigated the acute toxicity, developmental toxicity, neurotoxicity of the compound, and its effects on metabolism in vivo by dissolving the drug in an aqueous environment.

## 2. Results

### 2.1. Chemical Verification of Glabrene

In the pharmaceutical intermediate glabridin, impurities are often present, as shown in the HPLC profile (Figure 1), with a *t*_R_ of 8.10 min. In the sample we analyzed, the content of this impurity was 7.0% according to the area normalization method. Subsequently, we separated and purified the impurity components through HPLC. Its chemical structure was identified as glabrene by comparing the ^1^H and ^13^C NMR data (Figure S1 in the Supplementary Materials) with the reported data [19]. This compound is a characteristic component of licorice [19], and is a natural product classified as a prenylated isoflavonoid.

### 2.2. Developmental Toxicity Assessment of Glabrene

Zebrafish larvae are often used as high-throughput models to evaluate the biological activity and toxicity of known and new compounds due to their transparency, low dosage, short life cycle, high throughput, preservation of cell signals, and consistency with mammalian developmental phenotypes [20]. With the international trend toward eliminating animal testing, zebrafish larvae are a promising new method for whole organisms [21]. By employing this model, we conducted a thorough investigation into the potential adverse effects of glabrene exposure, thereby contributing to the safety evaluation of this botanical compound.

**Figure 2:**
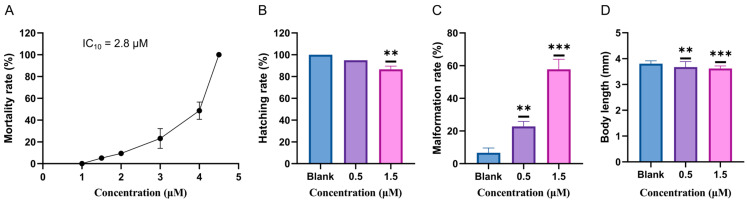
Acute toxicity assessment of glabrene on zebrafish larvae: (**A**) concentration–survival rate, with n = 3 and 30 larvae per replicate; (**B**) hatching rate, n = 3, 30 larvae in each replicate; (**C**) malformation rate, n = 30; (**D**) body length of zebrafish, n = 9. ** *p* < 0.01, *** *p* < 0.001.

The data in Figure 2A demonstrated a statistically significant, concentration-dependent increase in the mortality of zebrafish larvae upon exposure to glabrene. As the concentration of glabrene exposure increased, the mortality rate of zebrafish larvae gradually rose, particularly when the concentration exceeded 4.0 μM, at which point the mortality rate sharply increased. A concentration of 4.5 μM of glabrene resulted in the complete death of zebrafish larvae. Thus, the toxicity of glabrene exhibited a concentration-dependent relationship within the assayed range of 1.0 to 4.5 μM, with an estimated LC_10_ of 2.8 μM and an LC_50_ of 3.7 μM. We then assessed the glabrene toxicity on larvae development. Glabrene showed obvious toxicity to the growth and development of larvae at concentrations even lower than 2.0 μM. The hatching rate of 3 days post-fertilization (dpf) larvae declined to 86.7% when the larvae were exposed to 1.5 μM glabrene (Figure 2B). A low concentration (0.5 μM) of glabrene has a limited impact on the hatching rate (95.0%), showing no significant difference compared to the control group. Malformation rate and body length are representative phenotype indices for toxicity evaluation. Both 0.5 μM and 1.5 μM aggravated malformation (22.8% and 57.7%, respectively) but exhibited limited obstruction on body development (Figure 2C,D).

**Figure 3:**
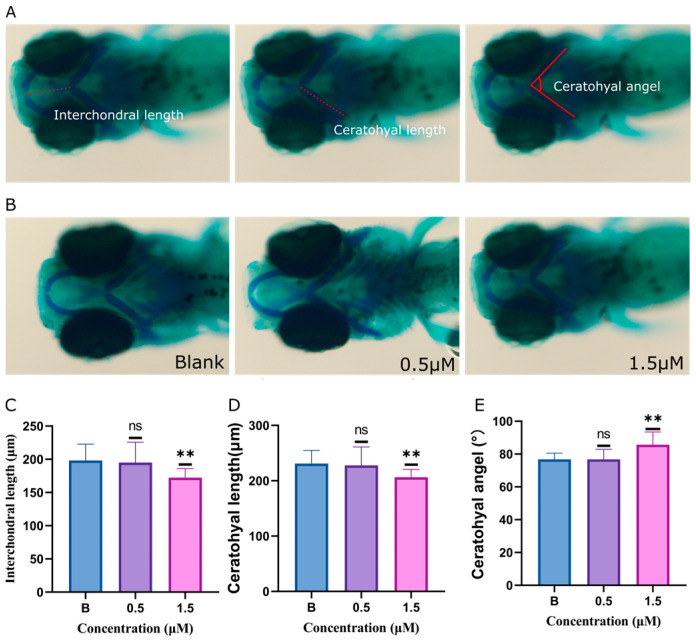
The influence of glabrene on head cartilage and vertebral mineralized bone development in zebrafish larvae. (**A**) A measurement pattern was established to assess the craniofacial structures. (**B**) Alcian blue staining was used to visualize the dorsal view of the zebrafish larval head. (**C**) The interval between Meckel’s cartilage and ceratohyal affected by glabrene exposure. (**D**) The length of the ceratohyal altered by glabrene treatment. (**E**) The angle of the ceratohyal affected by glabrene. Data are expressed as mean ± SD, n = 9; ns, not significant; ** *p* < 0.01.

Compared to zebrafish larvae exposed to 0.1% DMSO, zebrafish treated with 1.5 μM of glabrene exhibited changes in their cranial cartilage structure (Figure 3). Specifically, the distance between Meckel’s cartilage and the ethmoid plate decreased significantly. The distance in the blank control group and 1.5 μM glabrene-exposed groups were 198.2 μm and 172.4 μm, respectively. The length of the ethmoid plate shortened. The lengths of the blank control and 1.5 μM glabrene-exposed groups were 231.2 μm and 206.3 μm, respectively. The angle between the ethmoid plates increased. The average angles for the two groups were 76.7° and 85.7°, respectively. We examined the cartilage tissue development in zebrafish exposed to 0.5 and 1.5 μM glabrene. However, higher exposure concentrations resulted in mortality, making it challenging to observe the effects of higher exposure concentrations on cartilage tissue and developmental toxicity.

### 2.3. Behavioral Characteristics Intervented by Glabrene

**Figure 4:**
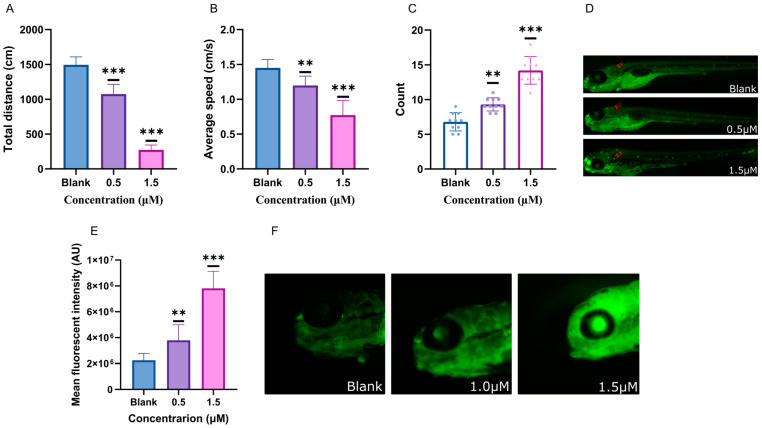
Neurotoxic effects of glabrene on zebrafish larvae. (**A**) Locomotor activity in zebrafish larvae exposed to glabrene. (**B**) Behavioral analysis of average speed. (**C**) Apoptosis in the nervous system of zebrafish larvae. (**D**) Fluorescent view of neural cell apoptosis. The red arrow points to the apoptotic nerve cells. (**E**) Quantification result of ROS levels in brain area. (**F**) Fluorescent view of ROS levels. n = 9, ** *p* < 0.01, *** *p* < 0.001.

In addition to the observed effects on body development, we also revealed that glabrene impacted the behavior of the zebrafish larvae. The total distances traveled, as depicted in Figure 4A, decreased by 28.2% and 81.6% when exposed to 0.5 μM and 1.5 μM of glabrene, respectively. In addition, the average speeds (Figure 4B) decreased by 17.3% and 46.6%, respectively. The decrease in physical ability followed a typical dose-dependent pattern. Based on the observed decrease in locomotor activity in zebrafish larvae exposed to glabrene, we hypothesized that the compound may cause damage to the nervous system. To observe the damage to the nervous system in zebrafish larvae, we used acridine orange staining to display the extent of nerve damage. After being stained, damaged nerve cells can be visualized as distinct fluorescent spots under a fluorescence microscope. Exposure to glabrene can significantly increase the fluorescent spots in the nervous system of zebrafish (Figure 4C,D). The number of spots in larvae after exposure to 1.5 μM glabrene was significantly higher than that exposed to 0.5 μM glabrene. The decrease in zebrafish locomotor behavior was consistent with the degree of damage to the nervous system. Furthermore, glabrene significantly increased the ROS levels in the brain (Figure 4E,F). Exposure to 0.5 and 1.5 μM of glabrene resulted in 1.7-fold and 3.5-fold blank levels in the zebrafish brain, respectively.

### 2.4. ACh, AChE, and Dopamine Levels Disturbed by Glabrene

**Figure 5:**
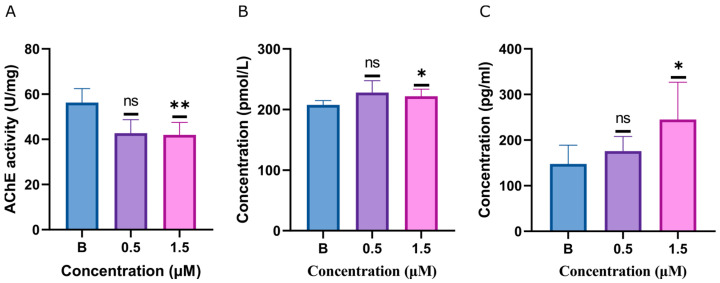
(**A**) AChE levels in zebrafish larvae. ACh (**B**) and dopamine (**C**) levels in zebrafish larvae. The data are presented as mean ± SD, n = 3, 50 larvae in each replicate; ns, not significant; * *p* < 0.05 vs. control, ** *p* < 0.01.

Behavioral disturbances caused by glabrene and indications from subsequent metabolomic analysis led us to further observe the impact of glabrene exposure on neurochemical levels in zebrafish. The data presented in Figure 5 reveal that the acetylcholinesterase (AChE) level in zebrafish larvae exposed to 0.5 μM glabrene did not differ significantly from the blank control group. A more pronounced effect was observed at the higher concentration of 1.5 μM glabrene. AChE level in the treated group decreased by 25.40% compared to the blank control. Additionally, the neurotransmitters acetylcholine (ACh) and dopamine levels were significantly elevated in the 1.5 μM glabrene-treated zebrafish larvae compared to the control group. Under exposure with 1.5 μM glabrene, the levels of ACh and dopamine increased by 6.84% and 65.93%, respectively.

### 2.5. Metabolome Variation Interfered by Glabrene

**Figure 6:**
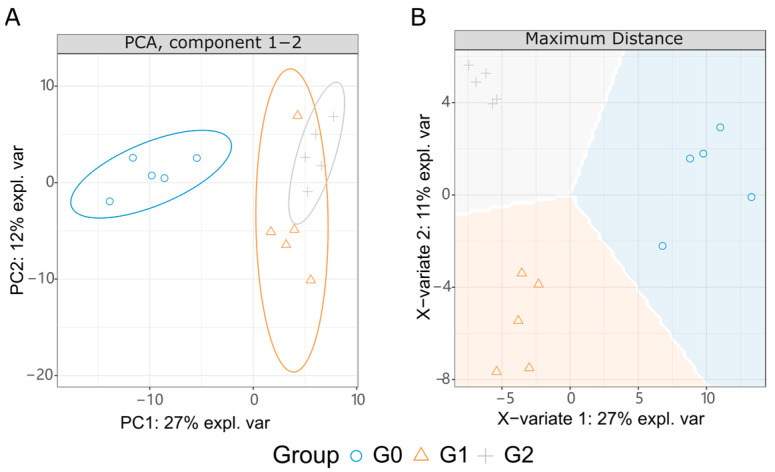
PCA (**A**) and PLS-DA (**B**) of zebrafish metabolism interfered by glabrene, n = 6. G0, blank control; G1, zebrafish exposed with 0.5 µM glabrene; G2, zebrafish exposed with 1.0 µM glabrene; QC, quality control.

To observe the interference of glabrene on normal metabolism in vivo, we further measured the metabolic profile in the zebrafish from the normal group (G0) and glabrene-exposed groups (G1 and G2). Glabrene exposure concentrations of G1 and G2 were 0.5 and 1.0 µM, respectively. The purpose of using two exposure concentrations was to facilitate the identification of metabolites that respond to glabrene variations in data analysis. Following scanning in LC-MS/MS positive and negative ion modes, we identified 209 endogenous metabolites. As shown in the principal component analysis (PCA) in Figure 6A, the comparative analysis of the metabolite levels revealed a significant difference between the glabrene-treated groups (G1 and G2) and the blank control group G0. The difference between the two exposed groups (G1 and G2) was limited. The partial least squares discriminant analysis (PLS-DA, Figure 6B) demonstrated distinct differences in metabolite levels and grouping. Identifying such differentially expressed metabolites could provide important insights into the metabolic pathways and physiological changes induced by the varying doses of glabrene exposure in the zebrafish larvae model.

**Figure 7:**
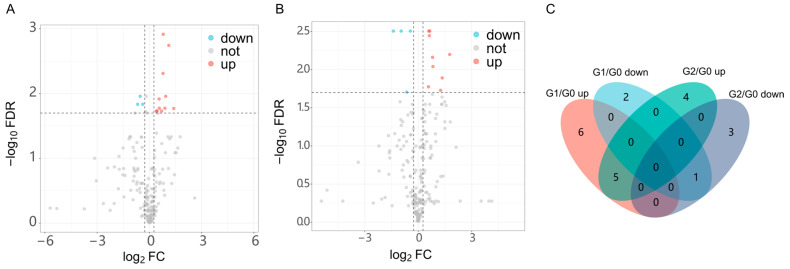
Metabolite variation in zebrafish exposed with glabrene, n = 5. (**A**) G1 vs. G0. (**B**) G2 vs. G0. (**C**) Metabolite counts in G1/G0 and G2/G1. Fold change > 1.2, adjusted *p* (FDR) < 0.02.

**Figure 8:**
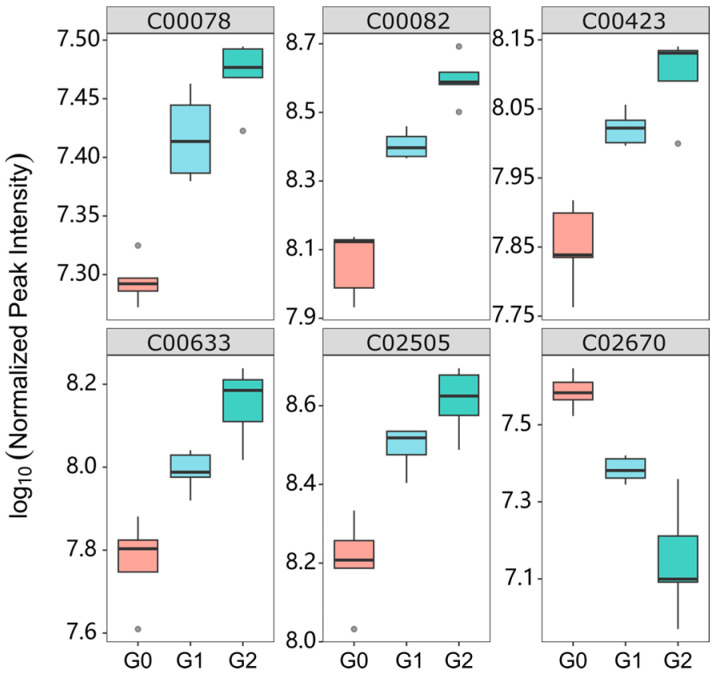
Differential metabolites interfered by glabrene, n = 5. C00078, KEGG entry number, L-tryptophan; C00082, L-tyrosine; C00423, trans-cinnamate; C00633, 4-hydroxybenzaldehyde; C02505, 2-phenylacetamide and C02670, D-Glucurone.

To identify key metabolites associated with glabrene treatment in zebrafish, we performed two comparisons, G1/G0 and G2/G0, based on PCA suggestions. In large-scale omics data analysis, correcting *p*-values is typically employed to mitigate the adverse effects of false positives. We utilized the false discovery rate (FDR) correction method in R base language. As shown in Figure 7A, 0.5 µM of glabrene upregulated 11 metabolites and downregulated 3 metabolites. In G2, 1.0 µM of glabrene (Figure 7B) upregulated nine and downregulated four metabolites. There were six common upregulated or downregulated metabolites in both exposed groups (Figure 7C and Figure 8), including upregulation of L-tryptophan (C00078), L-tyrosine (C00082), trans-cinnamate (C00423), 4-hydroxybenzaldehyde (C00633), and 2-phenylacetamide (C02505), as well as the downregulation of D-Glucuronic acid (C02670).

**Figure 9:**
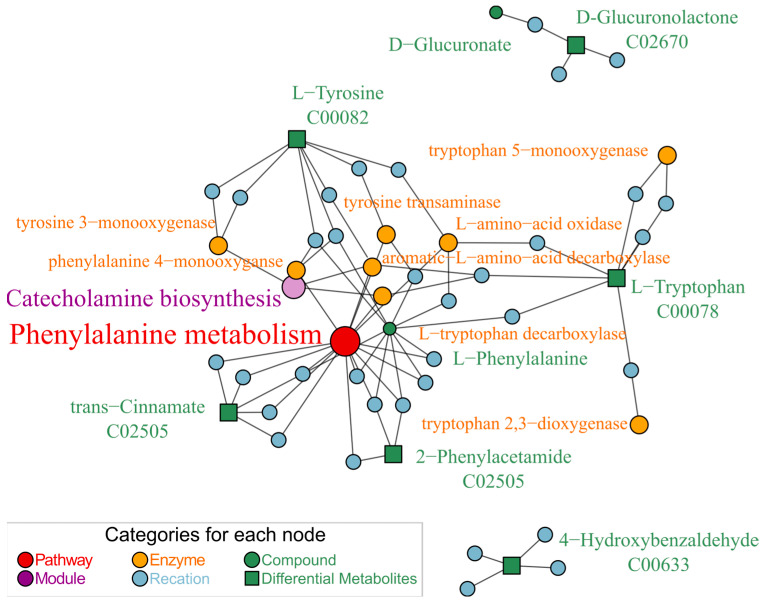
Glabrene-interfered metabolic pathway network, which was enriched and mapped by FELLA.

We conducted an enrichment analysis of metabolic pathways and biochemical reactions using FELLA (an R package in the Bioconductor community) to explore the biological information implied behind these key differential metabolites. The data analysis presented in Figure 9 demonstrates that all six identified differential metabolites were concentrated within specific metabolic reactions occurring in the zebrafish larvae. The enriched statistical *p* scores in Table S1 in the Supplementary Materials. Specifically, four metabolites were enriched on the catecholamine biosynthesis metabolic module (M00042) of the phenylalanine metabolism pathway (dre00360). In KEGG orthology, this metabolic module converts tyrosine into dopamine, noradrenaline, and adrenaline.

## 3. Discussion

### 3.1. Glabrene Exhibited Conspicuous Zebrafish Lethal Toxicity and Affected Body Development

Licorice (*G. glabra*) has a long history of culinary and medicinal uses across various cultures, making it a versatile and valuable ingredient in the food industry with various potential applications. Licorice root extract and its derivatives, such as glycyrrhizin and glycyrrhetinic acid, have been utilized as natural sweeteners and additives in the food and beverage industries [22]. For example, a mixture of glycyrrhetic acid glucuronide and glycyrrhizin can enhance flavors, eliminate undesirable tastes, and serve as a sweetening agent in various food products like ice cream, chocolate, soups, and beverages. Licorice is also an essential component of traditional Eastern medicines. Licorice and its derivatives are considered Generally Recognized As Safe (GRAS) substances by organizations like FEMA, the Council of Europe, and the United Kingdom, with specified limits on glycyrrhizin content to ensure safety.

Glabrene has been discovered in the roots of *G. glabra* and is known for its cytotoxic properties against cancer cells [23,24]. The presence of glabrene in the roots of *G. glabra* is significant as it serves as a distinctive chemical marker to differentiate Glycyrrhiza species and plays a crucial role in quality control for licorice products. Glabrene, along with other compounds such as glabridin and prenylated phenolic compounds, are considered important markers for identifying *G. glabra* roots. Glabrene also has been identified as a valuable compound in licorice roots, contributing to the plant’s economic value. This suggests that the presence of glabrene in the roots of *G. glabra* is indeed significant both for species identification and commercial purposes [23].

As a non-mammalian vertebrate, zebrafish is highly genetically similar to humans, with 87% of genes homologous to humans [25], including gene signaling pathways and physiological structure and function [26]. The bones [27], liver development, and nervous system [28] of zebrafish are highly similar to those of mammals. In addition, zebrafish and mammals have common developmental processes, such as neurodevelopment, axon formation, and related genetic signals, mammalian brain midbrain, diencephal, midbrain, etc.

While exploring the biological functions of glabrene using zebrafish larvae, we unexpectedly discovered the significant toxicity of glabrene. This compound caused 100% mortality in all tested zebrafish larvae at a low concentration of only 4.5 μM. This drove us to examine the toxic characteristics and mechanisms of this compound. This molecule is a representative compound in licorice [7] and a major impurity in the cosmetic raw material glabridin, a whitening ingredient widely used in cosmetics [29,30].

We selected zebrafish larvae to explore the toxicity features of glabrene since zebrafish are a valuable model for toxicology studies due to their small size, transparency during development, ease of maintenance, and shared genetic similarities with humans [31]. According to the survival curve of zebrafish larvae (Figure 2), the acute lethality rate LC_10_ of glabrebe was 2.8 μM. To facilitate the observation of the toxic characteristics of this compound, we exposed larvae to glabrene below the LC_10_, specifically at 0.5 and 1.5 μM. The careful observation of the zebrafish larvae exposed to the compound revealed a significantly increased incidence of developmental abnormalities compared to the control group. However, at the lower exposure concentrations examined, no obvious changes were detected in the hatching rate of the zebrafish eggs or the overall body length of the larvae.

Glabrene not only caused abnormalities in the development of larvae fish but also, to some extent, led to abnormalities in cartilage tissue (Figure 3). However, due to the acute toxicity of glabrene, we could only expose zebrafish to very low concentrations of glabrene (0.5 and 1.5 μM) in a living state. Once it exceeds 2.0 μM, zebrafish death occurred, affecting observations.

### 3.2. Glabrene Caused Nerve Damage, Disrupting the Balance of Neurotransmitters

Behavioral characterization of zebrafish can provide valuable insights into neurotoxicity induced by chemical substances. Abnormal behavior responses may indicate damage to the nervous system or neurobehavioral impairments, such as alterations in spontaneous swimming speed, response to stimuli, and social behaviors [32]. These changes in behavior serve as important endpoints in toxicological assessments and can help evaluate the potential neurotoxic effects of chemicals on aquatic organisms [33].

In the current behavior investigation, zebrafish exposed to glabrene significantly reduced the distance traveled and decreased average speed (Figure 4). Higher concentrations (1.5 μM) of glabrene lead to more pronounced decreases in the movement ability of zebrafish. Changes in locomotion behavior, such as decreased swimming activity, altered velocity, and reduced distance traveled, can indicate neurotoxicity caused by various environmental chemicals [31].

Apoptotic cells typically exhibit changes in their nuclear morphology, such as condensed or fragmented chromatin. Acridine orange can bind to DNA and RNA in these apoptotic cells, resulting in a change in fluorescence emission that can be visualized under a fluorescence microscope [34]. Fluorescence staining in the current investigation (Figure 4D,F) discovered an increase in necrotic cells in the zebrafish nervous system. This further proved that glabrene causes damage to the nervous system.

The levels of ROS in the central nervous system also increased (Figure 4E,F). All these findings can be attributed to central nervous system damage induced by glabrene. Flavonoids and flavonoid glycosides often possess effective properties in scavenging free radicals and reducing the level of ROS in the body due to their polyphenolic structure. This biological activity is crucial in maintaining overall health [35]. In our findings, glabrene, despite being a flavonoid substance, has a significant effect on increasing the levels of ROS in vivo. This could be an important factor contributing to the toxicity of glabrene.

### 3.3. Glabrene Affected the Metabolism of Phenylalanine In Vivo

Exposure of zebrafish larvae to glabrene led to level changes in metabolites L-tryptophan, L-tyrosine, trans-cinnamate, and 2-phenylacetamide. Enrichment analysis indicates that these metabolites were located on the catecholamine biosynthesis module of the phenylalanine metabolic pathway (Figure 9). This module is used to convert tyrosine into dopamine. The relationship between the phenylalanine metabolism pathway, dopamine, and neuronal damage can be linked through their involvement in various neurological processes. Phenylalanine is a start amino acid that plays a role in neurotransmitter synthesis, including dopamine, a crucial neurotransmitter associated with reward and movement control. Dysregulation in dopamine metabolism by enzymes (such as monoamine oxidase) can impact the bioenergetic needs of dopaminergic neurons, potentially affecting neurotransmitter release [36]. Dopaminergic neurons are vulnerable to oxidative stress and damage, which can be influenced by dopamine metabolism and its interactions with cellular redox status [37]. The phenylalanine metabolism pathway, dopamine, and neuronal damage could interconnect through their roles in neurotransmission, oxidative stress, and neurobiological processes within the brain. However, dopamine levels are precisely controlled by multiple key enzymes involved in its synthesis and metabolic pathways in the body. Further exploration is needed to understand how glabrene interferes with the activity of these enzymes.

### 3.4. Glabrene Interfered with Neurotransmitters

The motor deficits and metabolome variation prompted us to investigate the changes in larvae’s neurotransmitter levels after exposure to glabrene, including dopamine, acetylcholine, and acetylcholinesterase. Zebrafish larvae were exposed to 1.5 μM glabrene with increased dopamine and ACh levels and decreased AChE activity. This indicated neurobehavioral changes and imbalances in neurotransmitter levels [38]. When AChE activity is inhibited, it leads to the accumulation of ACh in the synaptic cleft [39]. Disruption or damage to the cholinergic system, dependent on ACh as the primary neurotransmitter, can lead to physiological changes and adverse effects in an organism [40]. Glabrene interfered with the motility of zebrafish larvae by downregulating AChE levels and induced ACh accumulation. Thus, glabrene caused zebrafish embryonic and larval developmental disorders and neurobehavioral alterations. Glabrene disrupted neurotransmitters by interfering with the cholinergic system, thereby reducing the locomotion capacity of zebrafish larvae. The intricate interplay between the two critical neurotransmitters, dopamine and ACh, regulates the integration of postsynaptic signaling within the striatal region. This antagonistic yet synergistic balance in stimulating striatal neurons underpins the functional, synergistic modulation of neurobehavioral processes in the zebrafish model organism. Through these refined dopamine–ACh interactions, any disruption to this delicate equilibrium may contribute to the observed alterations in the behavioral phenotypes of the treated zebrafish larvae [41]. The impairment of the cholinergic system can also result in aberrant dopaminergic neurotransmission, leading to the dysregulation of dopamine-related behavioral and physiological responses [42].

The relationship between neurotoxicity and oxidative stress, as mediated by ROS, in the context of AChE activity involves the impact of oxidative stress on the nervous system, particularly at the neuromuscular junction, where AChE plays a crucial role in regulating nerve impulse transmission [43]. The complex interplay between AChE, ROS, and neurotoxicity holds significant implications for developing treatments for neurodegenerative diseases. ROS can result in lipid peroxidation, impair neurotransmitter signaling, and oxidative damage to brain cells [44]. This oxidative damage is particularly detrimental to brain cells due to their high oxygen absorption. The accumulation of pro-oxidant compounds and reduced antioxidant mechanisms can lead to oxidative neurotoxicity, impacting neuronal functions and contributing to neurodegenerative diseases [45].

## 4. Materials and Methods

### 4.1. Chemicals and Materials

The dimethyl sulfoxide (DMSO) was acquired from Solarbio (Solarbio Life Sciences Co. Ltd., Beijing, China). Ultra-performance liquid chromatography (UPLC)-grade solvents were obtained from Fisher (Thermo Fisher Scientific Inc., Waltham, MA, USA). The adult zebrafish were procured from FishBio (Shanghai FishBio Co., Ltd., Shanghai, China). The water used in the experiments was sourced from A.S. Watson (A.S. Watson Group Ltd. Hong Kong, China).

### 4.2. Zebrafish Maintenance

The adult zebrafish were segregated by gender and maintained in fish culture water comprising 145 mg of sea salt per 1.0 L of water. The aquarium temperature was carefully regulated at 28.0 ± 0.5 °C, with a 14 h light and 10 h dark photoperiod. Additionally, the conductivity of the water was consistently monitored and maintained within the range of 480 to 510 μS/cm [46]. The adult zebrafish were fed a daily diet of fresh brine shrimp and commercial fish feed. Healthy adult zebrafish, comprising a 3:2 ratio of males to females, were randomly selected for breeding. The resulting embryos were collected after a 3 h incubation period. These embryos were then incubated in fresh E3 water at 28.0 ± 0.5 °C [47]. All procedures were approved by the Institutional Animal Protection and Utilization Committee of Northwest A&F University (Permit Number: XN2024-0403, approved on 23 July 2023).

### 4.3. Glabrene Separation and Purification

The raw glabridin material was provided by Huatai (Shaanxi Huatai Bio-fine Chemical Co., Ltd. Xi’an, Shaanxi, China) The raw glabridin (1.2 g) was de-pigmented on a Sephadex LH-20 column (MeOH), and then separated by reverse-phase C18 column chromatography with 80% acetonitrile elution to obtain crude glabrene. This crude product was further purified using an HPLC C18 column (250 × 10 mm, 5 μm, SilGreen, Beijing Greenherbs Technology Co., Ltd., Beijing China), which was eluted with 65% acetonitrile (2.0 mL/min) and inspected at 230 and 254 nm to yield glabrene (80.2 mg). The nuclear magnetic resonance (NMR) data were acquired using an AVANCE NEO 400 MHz instrument (Bruker Corporation, Fällanden, Zurich, Switzerland). The chemical structure of the compound was subsequently determined by comparing the obtained NMR data with the information reported in the literature [19].

Glabrene, white powder, ^1^H NMR (400 MHz, Acetone-d6) δ 7.05 (d, *J* = 8.5 Hz, 1H), 6.94 (d, *J* = 8.2 Hz, 1H), 6.69 (d, *J* = 10.0 Hz, 1H), 6.54 (s, 1H), 6.47 (d, *J* = 8.5 Hz, 1H), 6.41 (dd, *J* = 8.1, 2.4 Hz, 1H), 6.34 (d, *J* = 2.3 Hz, 1H), 5.66 (d, *J* = 10.0 Hz, 1H), 4.97 (s, 2H), 1.44 (s, 6H). ^13^C NMR (100 MHz, Acetone-d6) δ 158.98, 155.63, 153.88, 152.08, 129.30, 129.19, 128.79, 128.34, 121.07, 119.69, 117.82, 117.06, 110.20, 109.35, 108.78, 103.36, 76.88, 68.99, 27.88.

### 4.4. Acute Toxicity Assay on Zebrafish

The acute toxicity of zebrafish larvae was assessed according to the procedure in [48] with slight modification. Zebrafish larvae with 2dpf were used to evaluate acute toxicity. Samples were dissolved in DMSO to different concentrations, which were then diluted 1000 times with E3 water to achieve the desired exposure concentration (1.0–4.5 μM). No precipitate was observed due to the low concentration. Zebrafish larvae were cultured in the E3 water containing glabrene. The blank control was DMSO (0.1%, *v*/*v*). The culture water was replaced daily. After 72 h of exposure, the number of dead zebrafish larvae in each group was counted. Each experimental group consisted of 30 zebrafish larvae, and the experiment was repeated in triplicate. The toxicity of glabrene was evaluated based on the assessment of parameters such as the absence of heartbeat, embryonic coagulation, developmental abnormalities, and undescended tails [49]. The mortality rate was calculated using the following equation:

Mortality (%) = (Number of dead larvae/Total number of exposed larvae) × 100%

### 4.5. Development Toxicity Evaluation

The zebrafish larvae were observed using a Nikon fluorescence stereomicroscope SMZ25 (Nikon Corp., Tokyo, Japan). Healthy larvae at 2 dpf were selected and exposed to varying concentrations of glabrene (0.5 and 1.5 μM) until 5 dpf. The control group was treated with 0.1% DMSO. Each experimental group consisted of 20 zebrafish larvae, and the experiment was repeated in triplicate. Hatching was assessed on 3 dpf, and the malformation rate was determined on 5 dpf. Zebrafish larvae were considered abnormal if they exhibited any of the following characteristics, either individually or in combination: curvature of the spine, pericardial edema, absence of a swim bladder, or growth retardation [50]. The hatching rate and malformation rate were calculated using the following equations:

Hatching rate (%) = (Number of hatched larvae/Total number of exposed larvae) × 100%

Malformation rate (%) = (Number of malformed larvae/Total number of surviving larvae) × 100%

Zebrafish larval body length was determined by measuring the head-to-tail length [51]. Twenty zebrafish larvae and 3 replicates of each concentration were used for each concentration. The zebrafish larval body lengths were measured using ImageJ (Media Cybernetics, Bethesda, MD, USA).

### 4.6. Cartilage Tissue Observation

This observation was carried out following the protocol previously reported [52,53,54]. Zebrafish larvae (2 dpf) were divided into three groups (10 items in each group) and placed in a 6-well plate. The groups were exposed to 0.1% DMSO, 0.5 μM and 1.5 μM glabrene until 5 dpf, respectively. After exposure, the zebrafish were fixed in 4% paraformaldehyde for 4 h, washed with PBS three times, bleached with 3% H_2_O_2_ and 0.5% KOH, then rinsed with PBS three times. The cleaned zebrafish were stained with 0.1% Alcian blue for 12 h, dehydrated in acidified EtOH (5% HCl in 70% EtOH) three times, rehydrated in gradient concentrations of 75%, 50%, and 25% HCl-ethanol solutions, and finally rinsed with PBS three times before preservation in glycerol. The observation of the cartilage tissues was conducted using a Nikon fluorescence stereomicroscope SMZ25 (Nikon Corp. Tokyo, Japan) to measure distances between Meckel’s cartilage and the ceratohyal cartilage, ceratohyal cartilage length, and angle.

### 4.7. Neurometric Characterization Bioassay

Locomotor activity assays were performed according to the procedure in [55] with slight modifications. Zebrafish embryos at 2 dpf were randomly chosen and distributed into 6-well plates, with each well accommodating 30 specimens. A 3 mL dose of glabrene was introduced to every well, while 0.1% DMSO acted as the vehicle control. Subsequently, the plates were maintained in an incubator set at 28.0 ± 0.5 °C, subject to a diurnal cycle of 14 h of light followed by 10 h of darkness until the larvae reached 5 dpf. Upon the completion of the exposure period, the zebrafish larvae underwent triple rinsing with E3 medium and were subsequently stored in the same solution. Nine phenotypically normal larvae were arbitrarily selected and transferred to a 6-well plate containing 3 mL of E3 medium per well, ensuring ample space for free swimming. The plate was then positioned beneath a BC4K-36D2 model electron microscope (Dongwan Gopoint Co., Ltd., Dongwan, Guangdong, China) equipped with video recording capabilities. Following a 10 min acclimation period, larval movements were recorded for 30 min. The resulting footage was analyzed using Ethovision XT 8.0 software (Noldus Information Technology, Wageningen, Gelderland, The Netherlands).

To assess the impact of glabrene on neuronal apoptosis in zebrafish, we employed the acridine orange (AO) staining technique [56]. Following a 48 h exposure period, the larvae were subjected to three PBS washes. Subsequently, they were incubated with 5 mg/L AO for 30 min at 28 °C in darkness. After triple rinsing, the specimens were anesthetized using 0.04% tetracaine solution. Apoptotic brain cells were then visualized using a Nikon SMZ25 fluorescence stereomicroscope. The quantification of the bioassay results was performed using ImageJ.

To investigate the oxidative stress induced by glabrene in zebrafish larvae, we quantified ROS levels using DCFH-DA (Merck KGaA, Darmstadt, Germany) and measured mean fluorescent intensity [57]. Larvae exposed to various compound concentrations were immersed in a 20 μg/mL DCFH-DA solution and incubated for 60 min at 28.5 °C. Following incubation, the specimens were washed with fresh E3 medium and anesthetized using 0.04% tetracaine. A Nikon SMZ25 fluorescence stereomicroscope was employed to visualize the anesthetized larvae. ImageJ software subsequently analyzed the mean fluorescence intensity within the brain region.

### 4.8. AChE Level Analysis and Enzyme-Linked Immunoreaction Assay

To examine the effects on the zebrafish nervous system, we randomly selected 50 larvae at each exposure endpoint for AChE level quantification. Using an AChE activity assay kit (Solarbio Biotechnology Co., Ltd., Beijing, China), the larvae were homogenized in 0.9% saline, and the resulting supernatant was processed according to kit instructions. Absorbance measurements were taken at 412 nm using a Synergy HLx Microplate Spectrophotometer (Gene Co., Ltd., Hong Kong, China). AChE levels were then calculated based on protein concentration, which was determined using the provided assay kit. Additionally, ACh and dopamine levels were assessed following a previously reported protocol. An ELISA kit (Shanghai Xin Yu Biotech Co., Ltd., Shanghai, China) was employed to analyze changes in ACh and dopamine content within the zebrafish larvae.

### 4.9. Metabonomic Sample Preparation and Data Acquisition

We subjected 100 zebrafish larvae, aged 2 dpf, to varying concentrations of glabrene until they reached 5 dpf. A 0.1% DMSO solution served as the negative control. Post exposure, the larvae were washed, rapidly frozen in liquid N_2_, and lyophilized. Homogenization was performed using a JY92-IIDN cell disruptor (Ningbo Scientz Biotechnology Co., Ltd., Ningbo, China) at 30% power in MeOH, with the process conducted on ice. The homogenate was then centrifuged at 12,000 r/min for 20 min at 4 °C. The resulting supernatant underwent metabolomic analysis using a Thermo Orbitrap Exploris 120 LC-MS system (Thermo Fisher Scientific Inc., Waltham, MA, USA). Metabolite was separated on an Accucore AQ C18 column (1.9 μm, 150 × 2.1 mm, Thermos Fisher Scientific, Waltham, MA, USA). The data collection and analysis followed a previously reported dd-MS2 module protocol [58].

### 4.10. Statistical Analyses

Statistical analysis was conducted using the R scripting language. To assess the differences between the control and exposed groups, we employed a one-way analysis of variance (ANOVA) followed by Dunnett’s multiple comparisons tests.

## 5. Conclusions

In conclusion, a toxic impurity, glabrene, was identified from glabridin pharmaceutical raw materials and cosmetic raw materials derived from licorice root. The compound glabrene is also a natural component of *G. glabra*. In our investigation, glabrene demonstrated significant lethal toxicity to zebrafish with an LC_10_ of 2.8 μM. Although this compound has limited effects on the zebrafish egg hatching rate and body length, it notably increased the malformation rate and disrupted cartilage development in larvae at a concentration of 1.5 μM. Furthermore, glabrene led to a significant decrease in larval mobility and damaged brain neural tissues. The analysis of the internal metabolic pathways and quantification through the neurotransmitter ELISA indicate an abnormal activation of the phenylalanine metabolic pathway, resulting in elevated dopamine levels and increased ACh levels in vivo. Due to the cumbersome process of separating and purifying glabridin, it often contains impurities of glabrene. Therefore, our findings provide a new reference for enhancing the safety and quality control of licorice products.

## Data Availability

Data are contained within the article.

## Supplementary Materials

The following supporting information can be downloaded at https://www.mdpi.com/article/10.3390/ijms25168985/s1.
