# Supplementary material for: Toxic Features and Metabolomic Intervention of Glabrene, an Impurity Found in the Pharmaceutical Product of Glabridin
Source: Int J Mol Sci. 2024 Aug 18;25(16):8985. doi: 10.3390/ijms25168985 (PMC11354706; doi:10.3390/ijms25168985)
Supplement: Supplementary file 1 [file ijms-25-08985-s001.zip › ijms-3109614-supplementary.pdf]

Supplementary materials

Figure S1. ^{1}H (400 MHz) and ^{13}C (100 MHz) NMR spectrum of glabrene in acetone-d_6

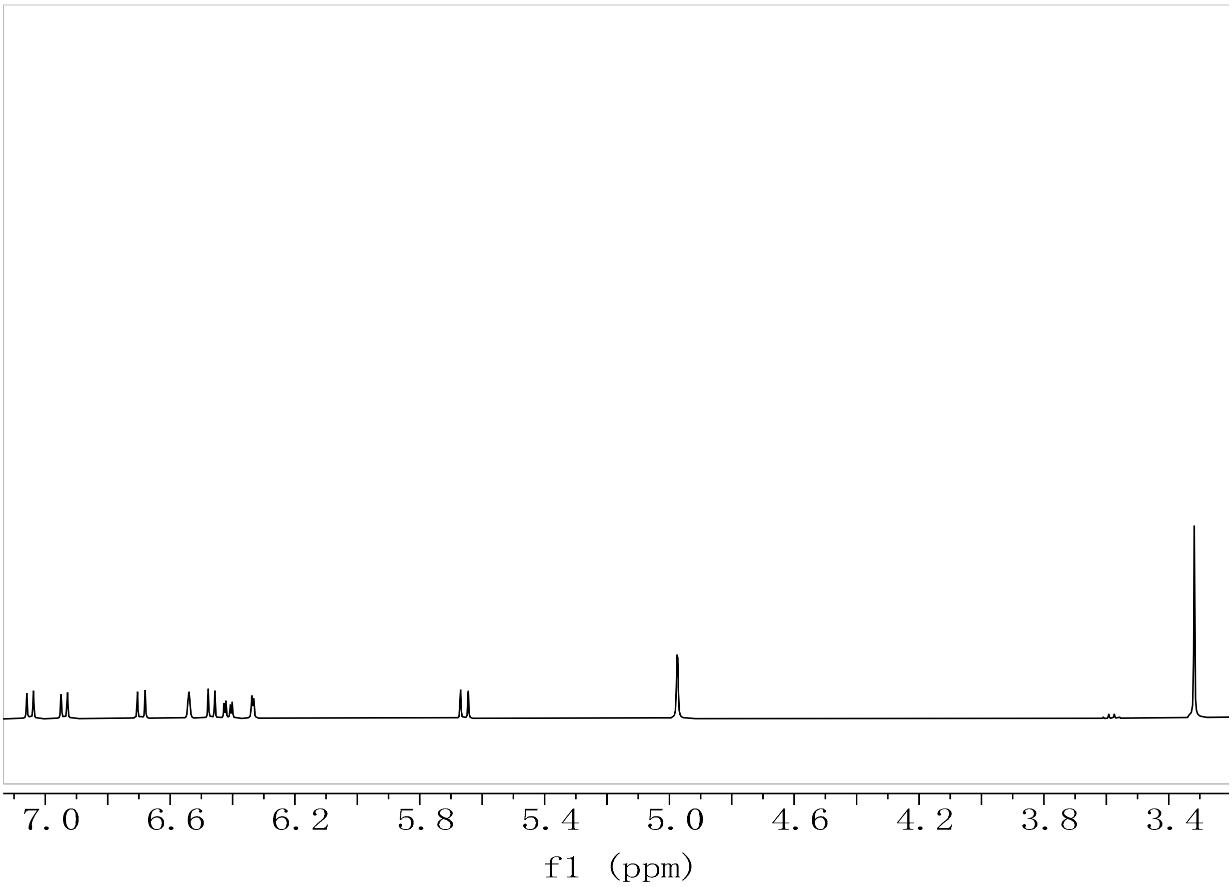

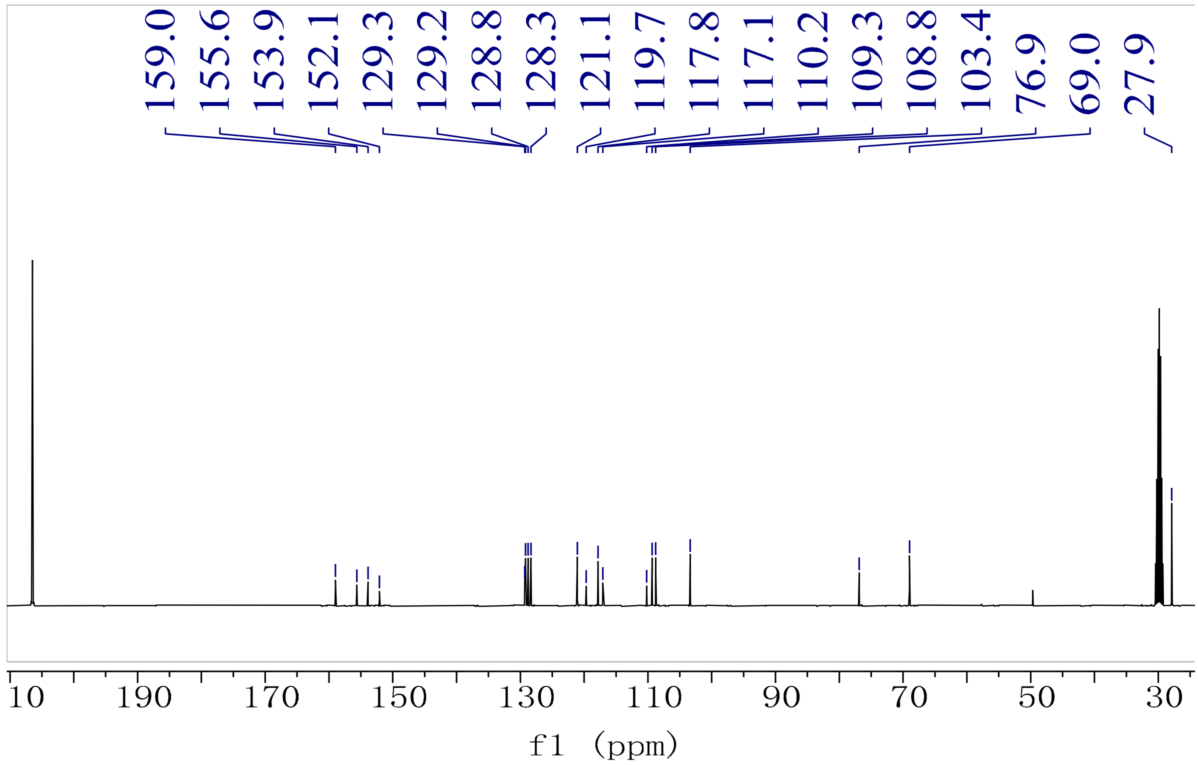
159.0
155.6
153.9
152.1
129.3
129.2
128.8
128.3
121.1
119.7
117.8
117.1
110.2
109.3
108.8
103.4
76.9
69.0
27.9
10
190
170
150
130
110
90
70
50
30
f1 (ppm)

Table S1. Enriched KEGG nodes by FELLA ($p < 0.01$)

KEGG ID	Entry type	KEGG name	P score
dre00360	pathway	Phenylalanine metabolism	1.00×10^{-6}
M00042	module	Catecholamine biosynthesis, tyrosine => dopamine => noradrenaline => adrenaline	9.87×10^{-6}
1.14.16.1	enzyme	phenylalanine 4-monooxygenase	1.00×10^{-6}
1.14.16.2	enzyme	tyrosine 3-monooxygenase	1.00×10^{-6}
1.14.16.4	enzyme	tryptophan 5-monooxygenase	1.00×10^{-6}
4.1.1.105	enzyme	L-tryptophan decarboxylase	1.00×10^{-6}
1.4.3.2	enzyme	L-amino-acid oxidase	7.21×10^{-6}
4.1.1.28	enzyme	aromatic-L-amino-acid decarboxylase	8.30×10^{-5}
2.6.1.5	enzyme	tyrosine transaminase	0.00014
1.13.11.11	enzyme	tryptophan 2,3-dioxygenase	0.0014
